# At the Crossroads of Lineage: Secondary Malignancies After CAR-Based Immunotherapy

**DOI:** 10.3390/cancers18040678

**Published:** 2026-02-19

**Authors:** Logan Lorentzen, Mazie Tsang, Talal Hilal, Allison Rosenthal, Javier Munoz

**Affiliations:** 1 Reno School of Medicine, University of Nevada, Reno, NV 89557, USA; 2 Division of Hematology and Oncology, Mayo Clinic Arizona, Phoenix, AZ 85054, USA; tsang.mazie@mayo.edu (M.T.); hilal.talal@mayo.edu (T.H.); rosenthal.allison@mayo.edu (A.R.); munoz.javier@mayo.edu (J.M.)

**Keywords:** CAR-T, DLBCL, T-cell lymphoma, BCMA, CD19, therapy-related myeloid neoplasm, second primary malignancy, long-term toxicity

## Abstract

CD19-directed chimeric antigen receptor (CAR) T-cell therapies have been instrumental in improving outcomes of refractory or relapsed B-cell malignancies. However, there have been safety concerns due to recent reports of second primary malignancies (SPMs) related to CAR T-cell therapies. We reviewed articles from Embase, PubMed, and Cochrane Library records and included SPM case reports as well as cohort studies. Across published cohorts, secondary cutaneous or peripheral T-cell lymphoma (PTCL) after diffuse large B-cell lymphomas (DLBCLs) have been reported at low incidence (generally in the low single-digit percentage range). While CAR T-cell therapy is associated with these rare secondary malignancies and lineage-switch events primarily described in acute leukemia, they are clinically significant and have resulted in increased surveillance. The currently available evidence suggests that most secondary malignancies after CAR T-cell therapy are due to background risk and prior treatment exposures rather than direct CAR T-cell therapy induced oncogenesis. However, rare CAR T-cell therapy-associated second primary T-cell malignancies have been reported. To properly define incidence, mechanisms, and risk factors for CAR T-cell therapy-associated malignancies, continued prospective registry follow-up and additional research will be needed.

## Simple Summary

Non-Hodgkin lymphomas (NHL), including diffuse large B-cell lymphoma (DLBCL), remain prevalent; hence, it is of utmost importance to find new treatments. Chimeric antigen receptor (CAR) T-cell therapy has shown tremendous promise in recent years. While this new therapy has shown admirable results, there have been clinically significant side effects, such as the emergence of secondary cancers following CAR T-cell therapy. Regardless of CAR T-cell therapy, patients with DLBCL are known to develop secondary malignancies, including T-cell lymphomas (TCL), in some rare cases. This review aims to summarize current evidence on reported second primary malignancies following CAR T-cell therapy.

## 1. Introduction

Diffuse large B-cell lymphomas (DLBCLs) are the most common non-Hodgkin lymphoma with hundreds of thousands of new cases diagnosed yearly [1]. This aggressive disease represents a significant burden, so finding new treatments to improve survival is critical. The traditional first-line treatment for DLBCL has remained curative for many patients with DLBCL with standard R-CHOP being a common therapy [2]. However, newer treatments like polatuzumab vedotin in addition to R-CHP have demonstrated improved patient outcomes compared to R-CHOP [3]. In high-risk activated B-cell/non-GCB subtypes such as double hit lymphoma, dose-adjusted etoposide, prednisone, vincristine, cyclophosphamide, and doxorubicin plus rituximab (DA-R-EPOCH) therapy is commonly used [4]. Despite all these advancements, many patients are refractory to this treatment or require additional lines of therapy [5,6].

A recent advancement that has impacted how lymphomas are treated is chimeric antigen receptor (CAR) T-cell therapy. While CAR T-cell therapy has expanded across hematologic malignancies, reports of second primary malignancies (SPMs) have showcased the need to understand the mechanisms underlying SPMs such as rare lineage-switch events reported mainly in acute leukemia. One such example was when a patient developed a fatal CD4-CD8 CAR-related PTCL 1 month after undergoing CAR T-cell therapy for relapsed primary central nervous system (CNS) DLBCL [7]. While such events are uncommon, this case still shows the importance of evaluating secondary T-cell malignancies that develop after CAR T-cell therapy. We aim to assess the available evidence regarding SPMs after CAR T-cell therapy with a focus on secondary T-cell lymphomas. We will also discuss competing mechanistic hypotheses and clinical implications for surveillance and molecular evaluation. It is important to note that epidemiological associations between BCL and TCL do not establish clonal transformation. Most CAR T-cell therapy secondary malignancies are also likely more indicative of increased baseline risk and prior treatment exposures rather than CAR T-cell therapy causing direct oncogenesis. In this review, we provide a focused overview of the second primary malignancies that have been reported following CAR T-cell therapy, particularly regarding TCL and available mechanistic evidence. The goal is to summarize current evidence, identify limitations, and knowledge gaps, and provide insights into what can be done in terms of surveillance and molecular evaluation when a secondary malignancy is suspected. This is of growing importance as CAR T-cell therapy continues to evolve and become more widespread.

## 2. Materials and Methods

This article is a narrative review. We searched Embase, PubMed, and the Cochrane Library and included relevant case reports, cohort studies, registry analyses, and review articles describing SPMs following CAR T-cell therapy. In this narrative review, we analyzed data from research articles covering SPMs arising after CAR T-cell therapy, including lymphomas and neoplasms beyond lymphomas, such as ALL, MM, MDS, and CLL. We included evidence from a variety of study designs, including multi-cohort studies, case studies, and clinical trials, to cover a broad range of the literature in the field. The case studies provided a more in-depth look into the mechanisms behind SPMs. In contrast, multi-cohort studies and clinical trials enabled the analysis of larger patient sample sizes, allowing for a more accurate assessment of the incidence of these events. We did not conduct any additional statistical analysis and only included those that were already present in the research articles.

Two reviewers independently performed a literature search. We searched various journal databases, including PubMed, Cochrane, and Embase, to conduct our literature search. Our inclusion criteria included case reports, multi-cohort studies, prospective studies, pharmacovigilance studies, clinical trials, and registries on SPMs that develop after CAR T-cell therapy, along with evidence that provided plausible connections between CAR T-cell therapy and the development of these SPMs. We also included research articles exploring the mechanistic links between TCL and DLBCL. Our exclusion criteria for research studies excluded non-human studies, research not in English, conference abstracts, editorials, commentaries, preprints, and gray literature.

## 3. Epidemiologic Association Between T-Cell Lymphomas and Diffuse Large B-Cell Lymphomas

Despite TCL and DLBCL being different lineages among aggressive non-Hodgkin lymphomas, there have been rare cases of patients with B-cell lymphomas who present with a composite T-cell lymphoma or vice versa. In 1993, Kim hypothesized that some composite lymphomas may be clonally related and thus represent stages of clonal evolution rather than separate lymphomas containing B-cell and T-cell components [8].

**Figure 1:**
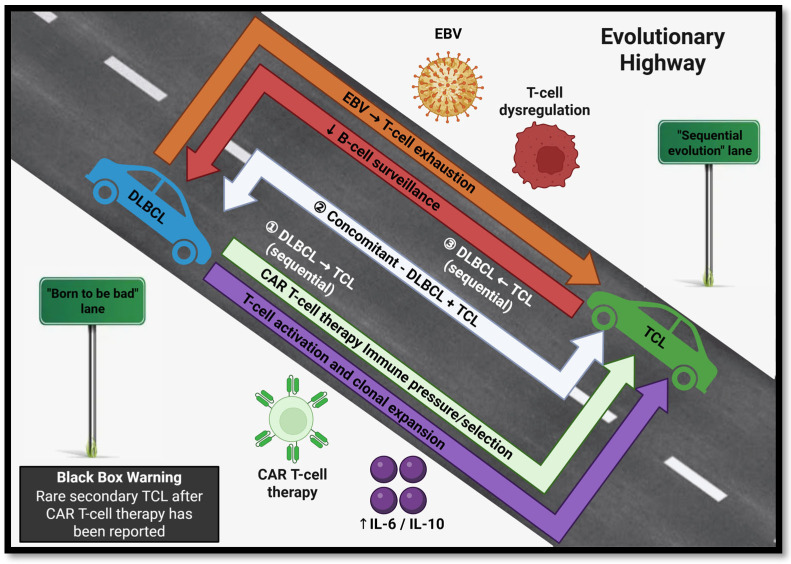
Conceptual framework illustrating hypotheses that may explain the observed epidemiologic association between DLBCL and TCL. This includes shared predisposition, immune dysregulation, treatment-related effects, and rare CAR T-cell therapy-associated oncogenesis effects. This schematic is not intended to imply clonal lineage-switch events as this requires molecular confirmation. Two conceptual lanes, “Born to be bad” and “Sequential evolution,” represent intrinsic predisposition versus environment-driven routes. The mechanisms shown include EBV-associated T-cell exhaustion and increased IL-6/IL-10 release-driven T-cell activation and clonal expansion (toward TCL), TCL-related dysregulation with decreased B-cell surveillance (toward DLBCL), CAR T-cell therapy immune pressure/selection, and a concomitant DLBCL + TCL pattern. A black box warning has also been included stating that “rare secondary TCL after CAR T-cell therapy has been reported.” Abbreviations: DLBCL, diffuse large B-cell lymphoma; TCL, T-cell lymphoma; EBV, Epstein–Barr virus; CAR T-cell, chimeric antigen receptor T-cell; IL, interleukin. Created with BioRender.com under a paid subscription license.

A more recent study from 2021 showed that the evidence for the relationship between DLBCL and TCL is not purely anecdotal but was also represented in population-level data. The study analyzed data collected in the population-based Surveillance, Epidemiology, and End Results (SEER) cancer registries, comprising 288,478 DLBCL and 23,747 TCL cases [9]. This analysis found that the risk of developing secondary TCL after a primary DLBCL diagnosis and vice versa was an almost fivefold increase compared to independently developing these conditions (standardized incidence ratio, SIR = 4.7 in both directions) [9]. Another important finding is that this increased risk persisted over five years, providing evidence that the increased risk is not due to surveillance bias or short-term treatment effects. The findings of this study showcase a bidirectional epidemiologic association between DLBCL and TCL. However, this association does not confirm clonal relatedness or transformation. This association may be due to a shared predisposition, immune dysregulation, treatment-related exposures, and/or surveillance effects (Figure 1).

A possible mechanism showcasing the bidirectional relationship between TCL and DLBCL is presented by a study investigating cytokine profiles in patients with DLBCL. In the study, 77 newly diagnosed patients with DLBCL had their cytokine levels assessed. The results found that the pretreatment had persistent secretion of IL-6 and IL-10, and that the levels of baseline IL-6 ≥ 4.5 pg/mL or IL-10 ≥ 5 pg/mL were indicative of being at a higher risk of relapse and worse survival. The increased levels of IL-6 and IL-10 secretion were also accompanied by increased levels of lactate dehydrogenase (LDH) and β2-microglobulin, showing the cancer was very active and had spread into bone marrow, causing symptoms [10]. Elevated inflammatory cytokines may be due to an immune-dysregulated tumor microenvironment and have been associated with prognosis in DLBCL. While these findings do not confirm that DLBCL can biologically give rise to TCL, they support the broader hypothesis that persistent immune activation and inflammatory signaling may assist in creating an environment that promotes the formation of secondary malignancies in susceptible patients.

Another possible mechanism that displays this bidirectional relationship is the Epstein–Barr Virus (EBV) acting as a connection between DLBCL and TCL. EBV has been implicated in select lymphoma types since EBV can contribute to immune dysregulation [11]. However, EBV is not universally present in sequential B-cell and T-cell lymphomas. Its role in secondary TCL is also not certain. EBV-related immune modulation is best considered a potential contributor to a subset of cases and not a unifying mechanism. Clonal hematopoiesis-associated mutations containing TET2 and DNMT3A have been reported in some patients with lymphoid malignancies and may reflect shared age-related predisposition rather than direct lineage-switching events [12]. Of note, these alterations are not specific to TCL as they have also been found to occur in myeloid malignancies. This supports a model of shared susceptibility rather than causal transformation (Figure 1).

## 4. Secondary Malignancies and Vector Integration

### 4.1. CAR T-Cell Therapy Transgene-Associated Malignancies

Vector integration has been proposed as a potential concern associated with the use of CAR T-cell therapy [13]. CAR T-cell therapy is created using retroviral or lentiviral vectors that insert their DNA into the DNA of host T-cells. Thus, this insertion could occur near an oncogene or tumor suppressor gene, potentially leading to the development of a secondary malignancy [13]. A case study showcasing this mechanism examined the development of an indolent TCL involving the small intestine. This malignancy occurred after treatment with ciltacabtagene autoleucel (cilta-cel) CAR T-cell therapy to treat the patient’s multiple myeloma (MM) [14]. The patient presented 4 months after the treatment with worsening non-bloody diarrhea and weight loss. Endoscopic examination revealed multiple duodenal ulcerations, and biopsy samples were then taken from these ulcers. CAR T-cell therapy transgene transcripts were found in the tumor cells through mRNA sequencing. Importantly, in situ hybridization for EBV was negative, and sequential sampling showed a persisting clonal T-cell arrangement. Whole genome sequences of the duodenal ulcers and peripheral blood found that a single lentiviral insertion site carrying the anti-BCMA CAR T-cell cassette was found within the second intron of the SSU72 gene. All these findings led the authors of the study to conclude that CAR T-cell therapy may have influenced the development of TCL due to the insertion event, with the timing of this development occurring shortly after CAR T-cell therapy, and the patient having no prior history of TCL. However, the authors also concluded that the development of TCL was not solely due to CAR T-cell therapy, given the presence of multiple other somatic alterations, such as copy-number variations and other mutations, that likely also influenced the development of TCL [14].

### 4.2. Second Primary Malignancies Without Detectable CAR T-Cell Therapy Transgene

Another study also detailed how vector integration influenced the development of a secondary malignancy after CAR T-cell therapy. Among 3066 patients treated with CAR T-cell therapy in the Database for the Evaluation of CAR T-cell therapy (DESCAR-T registry), only one patient developed a primary cutaneous CD30+ T-cell lymphoproliferative disorder [15]. This secondary malignancy developed three years after receiving tisagenlecleucel treatment for DLBCL. It was found through molecular analysis that the CAR clone from the CAR T-cell therapy had integrated into PLAAT4 (phospholipase A and acyltransferase 4), a tumor suppressor gene. While this study demonstrates an integration event, it occurred in only one out of 3066 patients, indicating a very low risk of developing T-cell malignancy after CAR T-cell therapy.

### 4.3. Clinical and Laboratory Evaluation When Secondary Malignancy Occurs

While previous studies have demonstrated vector integration, a case study has shown a clinical example of a patient developing a secondary malignancy in the absence of vector integration. This patient was one of 724 patients who had received CAR T-cell therapy at Stanford between 4 February 2016 and 15 January 2024, and was examined at their median 15-month follow-up appointment [16]. It was found that at these follow-up appointments, 25 SPMs in total had developed, with 14 of them being hematologic malignancies, 13 being acute myeloid leukemia or myelodysplastic syndrome, and the patient who is the focus of this case study was the only one who had developed a TCL. The patient was a 59-year-old who had received CAR T-cell therapy to treat a Stage IV EBV+ DLBCL that initially presented in her bone marrow and lymph nodes. After 54 days of axicabtagene ciloleucel (axi-cel) CAR19 therapy, the patient developed a separate TCL. The TCL had a distinct immunophenotype and genomic signature from the DLBCL [16]. However, there were some interesting similarities, such as sharing EBV positivity and association with DNMT3A/TET2 clonal hematopoiesis. One of the most significant findings from examining these neoplasms is the absence of evidence for oncogenic vector integration. It was suggested that the TCL may have occurred due to a pre-existing or pre-malignant clone because a T-cell clone was detectable at a low frequency (0.006%) before CAR T-cell infusion [16]. The findings of this study highlight the rarity of secondary TCL/DLBCL, as well as provide a framework for defining clonal relationships and monitoring viral vectors.

A study analyzing data from 38 clinical trials containing 783 patients also found evidence of secondary malignancies developing after CAR T-cell therapy. Among these 783 patients, 18 patients developed a secondary malignancy with a median onset of 1.94 years [17]. Seventeen of these secondary malignancies were myeloid disorders and solid tumors, and one was a TCL. Tumor samples were taken to evaluate any evidence of vector integration. The malignancies were found not to have any incidents of vector integration. The TCL not having evidence of vector integration indicates that it did not arise from T-cells modified by CAR T-cell therapy. Of the 783 patients, 176 had available integration site sequencing data. Analysis of the data revealed that while some insertions occurred near tumor suppressor cells, leading to clonal expansion, none of the insertions directly contributed to malignancy development [17].

In response to reports of TCLs following CAR T-cell therapy, the U.S. Food and Drug Administration (FDA) required class-wide boxed warning updates for CAR T-cell products and recommended that patients developing secondary malignancies be monitored lifelong. When secondary malignancies occur, the FDA recommends evaluation for the presence of CAR T-cell transgene and coordination with the CAR T-cell manufacturer to support appropriate testing and investigation [18]. Although such events appear to be exceptionally rare, they have important implications for post-treatment surveillance and standardized molecular evaluation.

## 5. Second Primary Malignancies in Neoplasms Beyond Lymphomas

Although this review focuses on secondary T-cell malignancies after CAR T-cell therapy, broader data on SPMs following CAR T-cell treatment provides essential context. Specifically, these studies help clarify overall incidence estimates, highlight that most SPMs appear related to prior therapy and baseline risk, and underscore the need for standardized molecular evaluation to identify rare CAR T-cell therapy-associated events. Secondary malignancies have also been associated with the use of CAR T-cell therapy in other diseases. In April 2024, following the receipt of more than 50 reports of secondary malignancies across all licensed CAR T-cell therapies, the United States Food and Drug Administration (FDA) added a boxed warning due to the reported secondary malignancies [19,20]. This indicates that secondary malignancies associated with CAR T-cell therapy are not limited to CAR T-cell therapies used in the treatment of lymphomas, but also encompass acute lymphoblastic leukemia (ALL), MM, and chronic lymphocytic leukemia (CLL) [21]. All these hematological diseases have been associated with therapy-related myeloid malignancies (t-MN) and lineage-switch events which have been reported in cases of acute leukemia, although it is essential to note that these complications remain relatively rare. An example of this mechanism was previously shown in the section describing vector integration events where a patient with MM developed indolent TCL after being treated with BCMA CAR T-cell therapy [14].

To explore these secondary malignancies after CAR T-cell therapy, published systematic reviews and meta-analyses have evaluated incidence patterns across disease types and follow-up durations. One of these studies analyzed 5517 recipients of CAR T-cell therapy who had been diagnosed with lymphoma or myeloma. The study’s results found that 326 patients presented with SPMs, with a median duration of 21.7 months [22]. Interestingly, myeloid SPMs were the most common, accounting for 56%, followed by new lymphoid malignancies [22]. While the risk of developing secondary malignancies is low, the fact that this risk is present for both CAR T-cell therapies targeting CD19 (for lymphoma/ALL) and BCMA (for myeloma) suggests that these malignancies may be influenced by prior chemotherapy exposure, particularly if vector integration is not present. To emphasize this point, only 326 out of 5517 CAR T-cell therapy patients developed secondary malignancies, indicating that this adverse event, although present, is not common and is likely due to factors other than the CAR T-cell therapy.

Another study investigating these secondary malignancies is the ELIANA trial. The ELIANA trial is a study evaluating the use of tisagenlecleucel, a drug developed to treat B-ALL in pediatric and young adult patients. The study comprised 79 patients with a median age of 11 years. The youngest patient was 3 years old, while the oldest patient was 23 years old. These patients received CD19 CAR T-cell therapy after fludarabine/cyclophosphamide lymphodepletion. The initial results of this study were very promising, with 81–82% of the patients having overall remission within 3 months of treatment [23]. However, the third-year follow-up for the ELIANA trial found that, across 79 patients, there was one case of lineage-switch acute myeloid leukemia (AML) and two cases of therapy-related myeloid neoplasms. In contrast, 59% of the patients remained relapse-free. These results yielded a cumulative incidence of approximately 3% across 3 years [23]. This provides further evidence that secondary malignancies arising from CAR T-cell therapy have occurred in other neoplasms beyond lymphomas. While the number of these secondary malignancies remains low, this result has been found in other patient cohorts, as was seen in a case study focusing on CD19 CAR T-cell therapy, which found that CD19-targeted immune pressure from this therapy can cause a lineage-switch from B-ALL cells to AML-like cells under CD19-directed selective pressure, including loss of CD19 surface protein [24]. This process was seen most prominently in leukemias containing *ZNF384* or *KMT2A* fusions [24]. The findings from these studies demonstrate that the selective loss of CD19 antigen in B-ALL cells enable these cells to transition into AML-like malignancies when subjected to selective pressure from CD19 CAR T-cell therapy.

Information about secondary malignancies related to the use of CAR T-cell therapy in MM is unfortunately limited, but the studies that have been conducted still hold valuable insights. AML and myelodysplastic syndrome (MDS) are among the neoplasms associated with the use of cilta-cel and idecabtagene vicleucel (ide-cel), as demonstrated in several series and case reports [25]. Cilta-cel and ide-cel are BCMA-targeted CAR T-cell therapies used for refractory or relapsed MM. Cilta-cel and ide-cel have become prominent treatments for MM, underscoring the need to investigate neoplasms associated with these CAR T-cell therapies. One of these critical case studies detailed how a patient with MM who received cilta-cel developed AML 14 months later. This finding is confirmed to have arisen after treatment since the patient had bone marrow absent of AML changes before the administration of cilta-cel [26]. These cases, in conjunction with the meta-analysis above, suggest that although the risk is small, the same risk of developing secondary cancers is present within both CD19 and BCMA and not limited to just lymphoma CAR T-cell therapies.

The use of CAR T-cell therapy is more limited in CLL than in MM and ALL at this point, resulting in less available study data compared to the previously discussed secondary malignancies. However, interest in this treatment has increased since lisocabtagene maraleucel (liso-cel), a CD19 CAR T-cell therapy, received accelerated FDA approval in March 2024 for the treatment of CLL [27]. This approval came with a class-wide boxed warning of the risk of cytokine-release syndrome, neurotoxicity, and, most importantly in terms of our analysis, secondary hematologic malignancies. Although disease-specific incidence data for CLL are not available, the FDA Adverse Event Reporting System has recorded 536 reports of SPMs [21]. This accounted for only 4.3% of all CAR T-cell therapy adverse event submissions and primarily comprised T-cell and myeloid cancers [21]. The data from this article, along with the previously discussed meta-analysis, demonstrate how secondary malignancies associated with CAR T-cell therapy occur across the full spectrum of B-cells and CLL. The table below summarizes the information previously reported (Table 1). 

## 6. Discussion

Second primary malignancies (SPMs) that occur after CAR T-cell therapy are rare events. However, understanding the mechanisms behind these occurrences is essential for properly discussing the potential risks to patients and for effectively monitoring patients for signs of these SPMs. Most SPMs across trials, pharmacovigilance data sets, and registries are myeloid neoplasms. Secondary TCLs after CAR T-cell therapy and lineage-switch events (primarily discussed in acute leukemia) are relatively rare and have been the subject of in-depth case studies. Reviewing the literature documenting these cases indicates that the absolute risk of these events remains low; however, the presence of these SPMs makes it crucial for the mechanisms leading to their development to continue being reported and analyzed.

Analyzing different multi-center cohort studies reveals consistent patterns behind the development of these SPMs. Myeloid SPMs were found to be most common after cytotoxic exposure and, in some cases, were preceded by evidence of clonal hematopoiesis, as shown in the Liu et al. article, with the presence of DNMT3A/TET2 clonal hematopoiesis in DLBCL, which are the same epigenetic modifiers that initiate follicular helper T-cell (TFH) type lymphomas [12]. A study involving multiple treatment centers found that lineage switching in acute leukemia may allow relapse with presentation as a different cancer [28]. Lastly, the development of SPMs was found to be mostly unaffected by vector integration [16,17]. In cases where vector integration was confirmed, the SPMs could not be definitively attributed to vector integration due to the presence of other risk factors [14,15]. These findings indicate that a patient’s treatment history is a risk factor for developing SPMs.

Mechanisms have been proposed that link CAR T-cell therapy and host factors to the development of SPMs beyond vector integration. One of these mechanisms is the expansion of already present clonal hematopoiesis due to inflammatory stress or cytopenic recovery caused by the increased release of inflammatory cytokines, such as IL-6 and IL-10, as observed in the study of 77 newly diagnosed patients with DLBCL [10]. The increased release of IL-6 and IL-10, along with the presence of other cofactors such as EBV, is proposed to create an environment that activates nearby T-cells. These mechanisms demonstrate that factors other than vector integration can lead to the development of SPMs, highlighting the importance of considering a patient’s treatment history when SPMs arise.

In addition to baseline inflammatory signaling observed in aggressive B-cell malignancies, CAR T-cell therapy is characterized by acute immune activation, most notably cytokine release syndrome (CRS), and this is typically IL-6-dominant. Inflammatory signaling that is driven by IL-6 have been shown to promote cellular proliferation, angiogenesis, and immune dysregulation through STAT3 and NF-kB pathways [29]. Also, sustained IL-6 signaling can contribute to chronic inflammatory states that facilitate genomic instability and malignant progression [30]. While CRS is transient, it represents a period of profound immune and inflammatory stress. This inflammation may theoretically promote expansion of pre-existing malignant or pre-malignant clones [31]. It is important to note that the available evidence does not support CRS as a direct oncogenic driver. CRS may instead function as an amplifier of background risk [32]. This hypothesis remains speculative and warrants further study.

The information gleaned from these studies has important implications in patient care. It highlights the importance of discussing the risk of SPMs with patients, emphasizing that while these events are rare, there is still a risk of them developing. SPMs being shown to arise from different risk factors presents the idea that baseline risk profiling, such as examining previous therapy exposures and CH testing when appropriate, may be necessary to calculate the risk of SPM development accurately. Since cytopenia was shown to be an early clinical sign in the development of SPMs, surveillance for the emergence of new or persistent cytopenia could lead to earlier intervention in treating SPMs [9]. The findings from these studies have had important implications in the usage of CAR T-cell therapy in patient care. Due to the potential risks of T-cell malignancies, the FDA made it a requirement that CD19 and BCMA CAR T-cell therapy products have a class-wide boxed warning. This warning included that the patients who receive these CAR T-cell therapy products should have lifelong monitoring [33].

A final implication is the collection of samples of SPMs, which can be used for integration site analysis and viral testing. Being able to perform these analyses on more tissue samples would allow for a greater number of SPMs to be analyzed and lead to a better understanding of their mechanisms. Informing the broader public of these findings is also essential. The current evidence suggests that most reported second primary malignancies following CAR T-cell therapy are more consistent with baseline risk factors and prior treatment exposures than with direct vector-related oncogenesis. However, CAR T-cell therapy-associated T-cell malignancies have rarely been reported [15].

Finally, while a narrative approach enables the synthesis of findings from various sources, it still has some inherent limitations. Some of these limitations include the potential for publication and reporting biases, the inability to report incidence estimates for all studies due to variable follow-up, and that the sources analyzed in this review use different forms of evidence, making it difficult for the data to align cleanly. The findings from the current literature suggest that SPMs that develop after CAR T-cell therapy are rare and that many may be due to baseline risk and prior treatment exposures. However, reporting bias, variable follow-up durations, and inconsistent molecular testing limit causal inference [20,34,35]. Continued prospective registry follow-up with standardized genomic sequencing will be needed to more accurately define incidence and mechanisms.

## 7. Future Directions and Conclusions

Second primary malignancies (SPMs) that develop after CAR T-cell therapy are rare, with myeloid neoplasms being the most common, and T-cell malignancies and lineage-switch events (mainly described in acute leukemia) being even rarer. Most cases of SPMs that have been tested have been found not to have any vector integration events present. They were found to have been caused instead by their therapy history and the overall health of the patient, rather than being a direct result of CAR T-cell therapy. Since CAR T-cell therapy has been shown to provide substantial and long-lasting benefits, its benefit-to-risk profile is very favorable.

Looking ahead, the current literature in the field has highlighted the need for prospective registries that have a standardized set of rules for data collection and require samples to be taken of all SPMs when they arise. These implementations would enable more SPMs to undergo integration site mapping, as well as viral testing, thereby increasing the amount of data available on the mechanisms leading to the development of SPMs. Other studies could also be conducted, such as longitudinal mechanistic studies to track the development of viral cofactors, including EBV, clonal hematopoiesis, and changes in the T-cell microenvironment, before and after the administration of CAR T-cell therapy. Clinically, there may be increased complete blood count (CBC) and bone marrow monitoring to watch for the development of new or persistent cytopenia. This would enable the earlier detection of SPMs, particularly in patients with prior cytotoxic exposure and clonal hematopoiesis. While CAR T-cell therapy provides substantial and durable benefit in otherwise lethal malignancies, there are still some possible improvements that could be made to reduce potential risk, such as utilizing virus-free editing and more targeted integration to avoid possible vector integration SPMs.

## Figures and Tables

**Table 1:** Reports of secondary malignancies outside of lymphoma after CAR T-cell therapy.

Disease/Indication	CAR T-Cell Product(s) (Targets)	Secondary Malignancies	Incidence or Time-to-Event	Key Source(s)
B-cell lymphoma + MM (pooled)	CD19 and BCMA products (mixed)	Mostly therapy-related myeloid neoplasms; some lymphoid and solid tumors	6.0% overall at median 21.7 months	[22]
Pediatric/young adult B-ALL	Tisagenlecleucel (CD19)	1-lineage-switch AML; 2 t-MDS/AML	3-year cumulative incidence = 3%	[23]
B-ALL case reports	Various CD19 products	Lineage-switch AML in ZNF384 and KMT2A fusions	Months to less than one year	[24]
Multiple myeloma	Ciltacabtagene autoleucel (Cilta-cel), Idecabtagene vicleucel (ide-cel) (BCMA)	t-MDS/AML; single AML after 14 months (case)	10% (4/40) in 28-month cohort; single AML at 14-month case report	[25,26]
Chronic lymphocytic leukemia	Lisocabtagene maraleucel (liso-cel) (CD19)	Myeloid and T-cell neoplasms (FAERS)	Too early for formal incidence; 536 CAR T-cell SPMs in FAERS (CLL now included)	[21]

Abbreviations: FAERS = Food and Drug Administration Adverse Event Reporting System; t-MDS/AML = therapy-related myelodysplastic syndrome/acute myeloid leukemia; SPM = second primary malignancy.

## Data Availability

No new data were created or analyzed in this study.
